# Ferrostatin-1 alleviates lipopolysaccharide-induced cardiac dysfunction

**DOI:** 10.1080/21655979.2021.2001913

**Published:** 2021-11-25

**Authors:** Zheng Xiao, Bin Kong, Jin Fang, Tianyou Qin, Chang Dai, Wei Shuai, He Huang

**Affiliations:** a Department of Cardiology, Renmin Hospital of Wuhan University, Wuhan, China; b Cardiovascular Research Institute of Wuhan University, Wuhan, China; c Hubei Key Laboratory of Cardiology, Wuhan, China

**Keywords:** Ferroptosis, inflammation, ferrostatin-1, cardiac dysfunction

## Abstract

Cardiac dysfunction is a common complication of sepsis, and is attributed to severe inflammatory responses. Ferroptosis is reported to be involved in sepsis-induced cardiac inflammation. Therefore, we speculated that ferrostatin-1 (Fer-1), a ferroptosis inhibitor, improves cardiac dysfunction caused by sepsis. An intraperitoneal injection of lipopolysaccharide (LPS) was performed to induce a rat cardiac dysfunction model. Echocardiography, cardiac histopathology, biochemical and western blot results were analyzed. Twelve hours after the LPS injection, LPS-treated rats exhibited deteriorating cardiac systolic function, increased levels of cardiac injury markers and levels of ferroptosis markers prostaglandin endoperoxide synthase 2 (PTGS2). Additionally, LPS increased iron deposition in the myocardium, with downregulating ferroportin (FPN, SLC40A1) and transferrin receptor (TfR)expression, and upregulating ferritin light chain (FTL) and ferritin heavy chain (FTH1) expression. Meanwhile, LPS also increased lipid peroxidation in the rat heart by decreasing the expression of glutathione peroxidase 4 (GPX4). Moreover, the expression of inflammatory cytokines, such as tumor necrosis-alpha (TNF-α), interleukin-1 (IL-1β), and interleukin-6 (IL-6), and inflammatory cell infiltration were also increased following LPS challenge. Finally, the abovementioned adverse effects of LPS were relieved by Fer-1 except for TfR expression. Mechanistically, Fer-1 significantly reduced the levels of toll-like receptor 4 (TLR4), phospho-nuclear factor kappa B (NF-κB), and phospho-inhibitor of kappa Bα (IκBα) in LPS-treated rats. In summary, these findings imply that Fer-1 improved sepsis-induced cardiac dysfunction at least partially via the TLR4/NF-κB signaling pathway.

## 1. Introduction

Sepsis is characterized by a severe inflammatory response leading to multiorgan failure [1]. In particular, cardiac dysfunction is an identified serious complication of sepsis, and it is associated with higher mortality rates and a poor prognosis [2,3]. Sepsis-induced systemic inflammatory responses, including cardiac inflammation, have been proposed to be involved in ferroptosis [4,5]. Importantly, an increasing number of studies have also reported an important role for ferroptosis in the pathogenesis of cardiac dysfunction [6,7]. However, the mechanism of ferroptosis-mediated, sepsis-induced cardiac dysfunction is still not fully understood.

Ferroptosis is an iron-dependent form of regulated cell death that is induced by the production of large amounts of lipid peroxidation products and abnormal iron metabolism [8,9]. Notably, glutathione peroxidase 4 (GPX4), ferroportin (FPN, SLC40A1), and transferrin receptor (TfR) are potent components that regulate ferroptosis, among which GPX4 is a lipid repair enzyme that protects cells from ferroptosis [10]. FPN, the sole known mammalian transmembrane iron export protein, regulates intracellular iron release to the extracellular space [11]. The function of TfR is to increase iron uptake, which in turn increases the intracellular iron level [11]. Most intracellular iron is stored in ferritin, which is composed of both ferritin light chain (FTL) and ferritin heavy chain (FTH1) subunits [11]. Additionally, a recent study found that prostaglandin endoperoxide synthase 2 (PTGS2) serves as a downstream marker of ferroptosis and promotes the secretion of inflammatory molecules [10]. With the development of ferroptosis, affected cells die and release damage-associated molecular patterns (DAMPs) and alarmins, most of which induce inflammatory responses by signaling through the Toll-like receptor 4 (TLR4) [8,12]. Specifically, TLR4 activation leads to the transfer of nuclear factor kappa B (NF-κB) from the cytoplasm to the nucleus and the subsequent expression of a series of inflammatory cytokines, such as tumor necrosis-alpha (TNF-α), interleukin-1 (IL-1β), and interleukin-6 (IL-6) [13]. Furthermore, TLR4/NF-κB signaling has been shown to play a role in promoting sepsis-induced cardiac dysfunction [14]. Therefore, inhibiting ferroptosis in the myocardium might be an effective approach to protect against cardiac inflammation.

Ferrostatin-1 (Fer-1), a synthetic compound isolated from small molecule libraries, is reported to reduce lipid peroxidation and inhibit ferroptosis [15]. In addition, Fer-1 also exerts an effective anti-inflammation effect; for example, acute kidney injury-associated production of inflammatory cytokines is decreased in Fer-1-treated mice [16]. Similar results were observed in a hepatic ischemia reperfusion injury model [17]. In a myocardial infarction model, Fer-1 protects the heart from inflammatory damage, thereby improving cardiac function and reducing interstitial fibrosis [18]. However, few studies have assessed on whether Fer-1 alleviates sepsis-induced cardiac dysfunction.

The present study aims to investigate the effects of Fer-1 on LPS-induced cardiac dysfunction and whether Fer-1 exerts its function in sepsis-induced cardiac dysfunction by inhibiting ferroptosis and inflammation, and modulating the TLR4/NF-κB signaling pathway.

## 2. Methods

### 2.1. Animals and treatment

Six-week-old healthy Wistar male rats were provided by SiPeiFu Biotechnology Co., Ltd. (Beijing, China) and raised at the Experimental Center of Wuhan Third Hospital. All animal procedures followed the Guide for the care and the Use of Laboratory Animals, issued by the National Institutes of Health in 1996. All rats were housed in a standard circumstance under controlled ambient temperatures, humidity, and dark/light cycles, and were provided free access to food and water.

### 2.2. Experimental protocols

To establish sepsis-induced cardiac dysfunction model, LPS was administered at a dose of 10 mg/kg by intraperitoneal injection as previously described [7]. Ferrostatin-1 (Fer-1, 10 mg/kg), a specific inhibitor of ferroptosis, was diluted with 0.01% DMSO in saline and intraperitoneally injected 1 hour before LPS administration. All rats were divided into four groups: Control, Fer-1, LPS and LPS+Fer-1 (n = 16 rats per group). Twelve hours after the LPS injection, cardiac tissues and serums were collected and stored at −80°C for biochemical studies.

### 2.3. Echocardiography

Twelve hours after the LPS injection, rats were lightly anesthetized with 1.5–2% isoflurane. A special ultrasound instrument (Vinno Technology, China) for small animals was used to obtain echocardiographic images as previously described [19]. The left ventricular ejection fraction (LVEF), left ventricular fractional shortening (LVFS), left ventricular end-systolic volume (LVESV), left ventricular end-diastolic volume (LVEDV), and left ventricular end-systolic posterior wall thickness (LVPWs) were recorded.

### 2.4. Biochemical and serum analyses

As described in a previous study, blood samples were collected and analyzed [19]. Serum levels of creatin kinase myocardial bound (CK-MB), lactate dehydrogenase (LDH), and aspartate aminotransferase (AST) were measured using an automatic biochemical analyzer (IDEXX laboratories Inc., Maine, USA). The serum levels of tumor necrosis-alpha (TNF-α, ELK1396, GM1150, Servicebio,ELK Biology, Wuhan, China), interleukin-1 (IL-1β, GM1153, Servicebio, Wuhan, China), and interleukin-6 (IL-6, GM1155,Servicebio,ELK1158, ELK Biology, Wuhan, China) were measured using commercial kits according to the instruction of manufacture.

### 2.5. Histological analysis

Using a previously described method, heart samples were collected, fixed with a 4% paraformaldehyde solution and embedded in paraffin. Several slices (4–5 mm thick) of the heart were prepared, and hematoxylin and eosin (H&E) staining was performed using a standard technical protocol [20]. All micrographs were acquired using a high-resolution optical microscope.

### 2.6. Prussian blue staining

Methods for Prussian blue staining have been described previously [7]. After paraffin embedding, the cardiac tissues were sliced into 4–5 mm thick sections and soaked in Perls’ staining solution (hydrochloric acid mixed with potassium ferrocyanide 1:1) for 10 minutes. The cardiac tissues sections were viewed using a high-resolution optical microscope.

### 2.7. Western blots

Methods for western blotting have been described previously by our laboratory [21]. In brief, total proteins extracted from frozen heart tissues were homogenized in the RIPA lysis buffer (Servicebio, Wuhan, China) containing various inhibitors, and protein concentration was quantified using a BCA Protein Assay Kit (Servicebio, Wuhan, China). The lysates were separated on 8–15% SDS-PAGE gels and then electro-transferred to 0.45 μm polyvinylidene difluoride (PVDF) membranes (Millipore, America). After blocking with 5% nonfat milk in Tris-buffered saline at room temperature for 2 h, the membranes were incubated for overnight at 4°C with the following primary antibodies: GPX4 (Abcam, ab125066), FPN (Abcam, ab58695), TfR (Invitrogen, JF0956), PTGS2 (CST, #12282S), FTH1 (CST, #3998), FTL (Abcam, ab69090), TNF-α (Proteintech, 17,590-1-AP), IL-1β (Abcam, ab9722), IL-6 (Abcam, ab9324), TLR4 (Affinity, AF7017), NF-κB p65 (CST, #8242S), P-NF-κB p65 (CST, #3031S), NF-κB p50 (Abcam, ab32360), IκBα (CST, #4814S), P-IκBα (CST, #2859S), and GAPDH (CST, #5174S). Subsequently, the membranes were incubated with an HRP-conjugated secondary antibody for 1 h at room temperature. The blots were imaged using a Bio-Rad imaging system (Bio-Rad, USA) and protein levels were determined using ImageJ software.

### 2.8. Statistical analysis

All data were analyzed using GraphPad Prism software. Student’s t test was applied to compare two groups, and the error bar represents the standard error of the mean (SEM). ANOVA was used to compare four or more groups, followed by the Bonferroni post hoc test. Categorical data are reported as percentages and were analyzed using Fisher’s exact test. P < 0.05 was considered statistically significant.

## 3. Results

The current study explores the effects of Fer-1 on sepsis-induced cardiac dysfunction and the underlying mechanism. We found that Fer-1 improved cardiac systolic function and alleviated cardiac injury. These effects of Fer-1 may be related to the decreases in intracellular iron accumulation and cardiac and serum inflammatory cytokines levels, which occurs at least partially through the inhibition of the TLR4/NF-κB signaling pathway.

### 3.1. Fer-1 improved systolic function and reduced the death rate of septic rats

**Figure 1.**
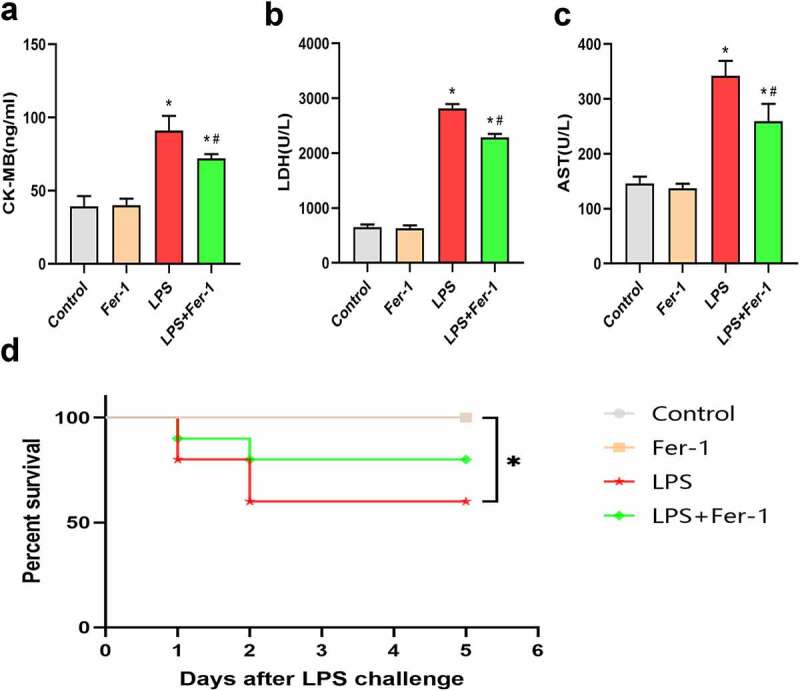
Fer-1 alleviated LPS-induced cardiac injury. (a-c) Representative myocardial enzymes and statistical analysis of expression of CK-MB, LDH and AST in four groups of rats (n = 3 per group). (d) Survival status of Control and Fer-1 group after LPS stimulation (n = 10 per group). Data are expressed as mean ±SEM. *P < 0.05 vs. Control group, **#**P < 0.05 vs. LPS group

Abbreviations: Fer-1, Ferrostatin-1; LPS, Lipopolysaccharide; CK-MB, Creatine kinase isoenzymes; LDH, Lactate dehydrogenase; AST, Glutamic oxalacetic transaminase.

We first assessed cardiac function using echocardiograph. As shown in Table 1, the LVEF (%) and LVFS (%) were decreased in the LPS group, and the decreases in these parameters were significantly inhibited by Fer-1. Moreover, the LVESV and LVEDV were increased in the LPS group, and these changes were significantly inhibited by the Fer-1 treatment. However, no difference in LVPWs was observed among the four groups. In addition, serum levels of various biomarkers, such as CK-MB, LDH, and AST, were measured to assess the severity of cardiac injury. As expected, the increased serum levels of CK-MB, LDH, and AST in the LPS group were significantly reduced by Fer-1 treatment (Figure 1(a-c)). After 5 days of LPS challenge, we found that Fer-1 reduced the mortality rate in rats, but the difference did not reach statistical significance (60% vs. 80%, P > 0.05; Figure 1(d)).

**Table 1.** The echocardiographic data of rats in groups

	Control	Fer-1	LPS	LPS+Fer-1
LVEF, %	86.19 ± 3.21	85.06 ± 3.84	72.09 ± 4.33*	78.60 ± 3.90 *#
LVFS, %	54.39 ± 2.26	54.05 ± 3.47	36.91 ± 4.50*	46.21 ± 4.83 *#
LVESV, ml	0.05 ± 0.02	0.05 ± 0.02	0.14 ± 0.04*	0.10 ± 0.04 *#
LVEDV, ml	0.27 ± 0.03	0.26 ± 0.04	0.42 ± 0.05*	0.36 ± 0.04 *#
LVPWs, mm	1.13 ± 0.27	1.14 ± 0.28	1.00 ± 0.29	0.99 ± 0.33

n = 8 per group. Data are expressed as mean ±SEM. *P < 0.05 vs. Control group, **#**P < 0.05 vs. LPS group.

Abbreviations: LVEF, left ventricular ejection fraction; LVFS, left ventricular shortening fraction; LVESV, left ventricular end-systolic volume; LVEDV, left ventricular end-diastolic volume; LVPWs, left ventricular end-systolic posterior wall thickness;

### 3.2. Fer-1 inhibited LPS-induced ferroptosis in the myocardium

**Figure 2.**
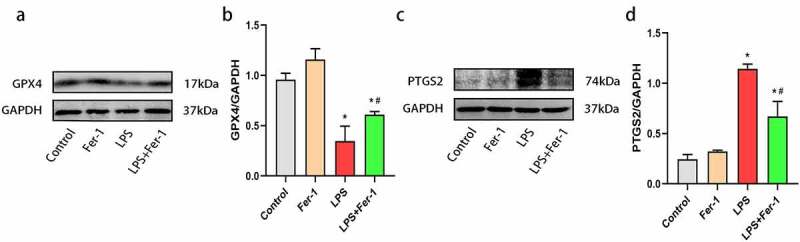
Fer-1 inhibited LPS-induced ferroptosis in myocardium. (a, b) Representative western blots and statistical analysis of expression of GPX4 in four groups of rats (n = 3 per group). (c, d) Representative western blots and statistical analysis of expression of PTGS2 in four groups of rats (n = 3 per group). Data are expressed as mean ±SEM. *P < 0.05 vs. Control group, **#**P < 0.05 vs. LPS group

Abbreviations: Fer-1, Ferrostatin-1; LPS, Lipopolysaccharide; GPX4, Glutathione peroxidase 4; PTGS2, Prostaglandin endoperoxide synthase 2.

**Figure 3.**
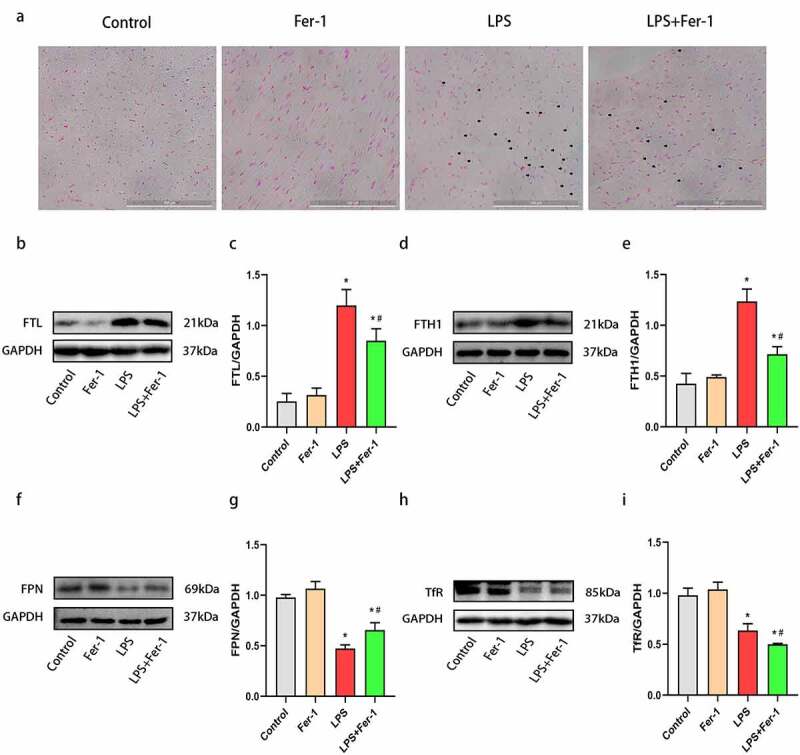
Fer-1 regulated LPS-induced iron overload in myocardium. (a) Prussian blue staining for iron respectively of rats in four groups (n = 3 per group). (b, c) Representative western blots and statistical analysis of expression of FTL in four groups of rats (n = 3 per group). (d, e) Representative western blots and statistical analysis of expression of FTH1 in four groups of rats (n = 3 per group). (f, g) Representative western blots and statistical analysis of expression of FPN in four groups of rats (n = 3 per group) (h, i) Representative western blots and statistical analysis of expression of TfR in four groups of rats (n = 3 per group). Data are expressed as mean ±SEM. *P < 0.05 vs. Control group, **#**P < 0.05 vs. LPS group

Abbreviations: Fer-1, Ferrostatin-1; LPS, Lipopolysaccharide; FTL, Ferritin light chain; FTH1, Ferritin heavy chain; FPN, Ferroportin; TfR, Transferrin receptor.

To confirm the role of ferroptosis in LPS-induced cardiac dysfunction, we examined the expression of the GPX4 and PTGS2 proteins. The GPX4 level was decreased after LPS administration, and the decrease was apparently inhibited by Fer-1 (Figures 2(a) and (b)). Next, we analyzed the levels of a key marker of ferroptosis, PTGS2, and observed increased expression of PTGS2 protein in the LPS group compared with the Control group. Fer-1 significantly reduced the LPS-induced increase in PTGS2 levels (Figures 2(c) and (d)). Moreover, we confirmed the remarkable accumulation of iron contents in the myocardium after LPS administration by performing Prussian blue staining, but the level of iron deposition was alleviated in the LPS+Fer-1 group compared with the LPS group (Figure 3(a)). Meanwhile, we detected increased levels of the FTL and FTH1 proteins after LPS administration, but Fer-1 significantly reduced the levels of these proteins (Figure 3(b-e)). Next, LPS downregulated the level of the iron export protein FPN, and these effects were partially eliminated by Fer-1 (Figures 3(f) and (g)). High expression of TfR indicates the increase of iron inflow level. Interestingly, LPS reduced the expression of the iron import protein TfR, and Fer-1 furtherly decreased the level of TfR, suggesting that the trend of iron inflow was further alleviated (Figures 3(h) and (i)). There data suggest that ferroptosis plays an important role in sepsis-induced cardiac dysfunction.

### 3.3. Fer-1 alleviated the inflammatory response in septic rats

**Figure 4.**
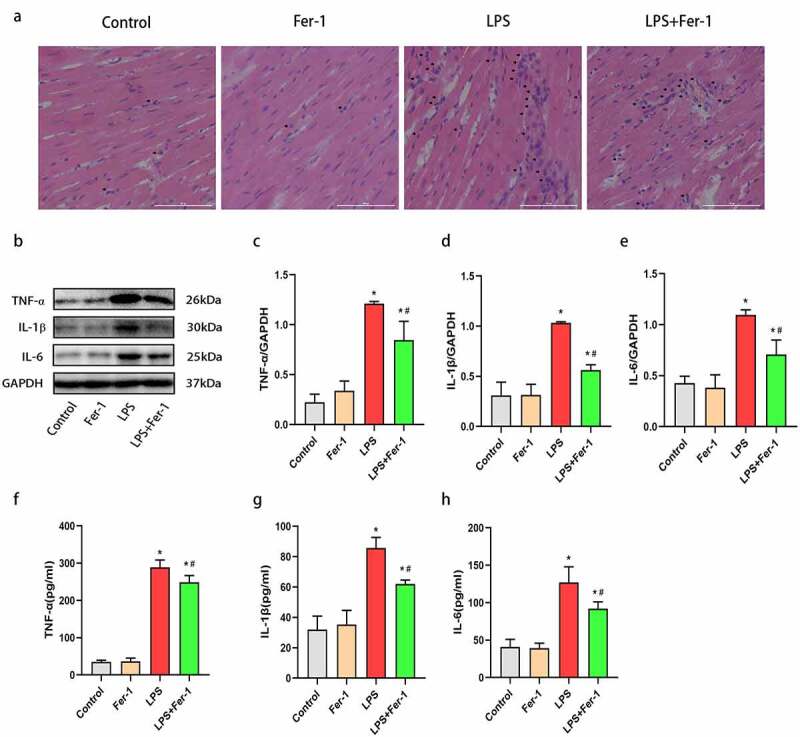
Fer-1 alleviated cardiac inflammatory response in septic rats. (a) Representative images of the morphological analysis and inflammatory cells infiltration as reflected by the H&E staining respectively of rats in four groups (n = 3 per group). (b-e) Representative western blots and statistical analysis of the TNF-α, IL-1β, and IL-6 protein levels in myocardium respectively of rats in four groups (n = 3 per group). (f-h) Representative enzyme-linked immunosorbent assay and statistical analysis of the serum TNF-α, IL-1β, and IL-6 levels respectively of rats in four groups (n = 3 per group). Data are expressed as mean ±SEM. *P < 0.05 vs. Control group, **#**P < 0.05 vs. LPS group

Abbreviations: Fer-1, Ferrostatin-1; TNF-α, Tumor necrosis factor-α; IL-1β, Interleukin-1; IL-6, Interleukin-6.

According to previous studies, inflammation contributes to the development of sepsis-induced cardiac dysfunction [5,14]. Therefore, we performed H&E staining to assess inflammatory cell infiltration in the heart. The level of inflammatory cell infiltration was increased in LPS-treated rat hearts, and this increase was apparently inhibited by Fer-1 (Figure 4(a)). Additionally, we also analyzed the levels of inflammatory cytokines (TNF-α, IL-1β, and IL-6) in the heart using western blotting. The expression of the TNF-α, IL-1β, and IL-6 proteins was upregulated after LPS administration, and the increases were prevented by Fer-1 (Figure 4(b-e)). Consistent with the western blotting results, enzyme-linked immunosorbent assays further revealed that Fer-1 significantly alleviated the systemic inflammation caused by LPS, as evidenced by the decreased the serum TNF-α, IL-1β, and IL-6 levels (figure 4(f-h)). Based on these findings, Fer-1 alleviates inflammatory responses during sepsis.

### 3.4. The levels of TLR4, NF-κB p50, P-NF-κB p65/NF-κB p65, and P-IκBα/IκBα were decreased by Fer-1 in the hearts of septic rats

**Figure 5.**
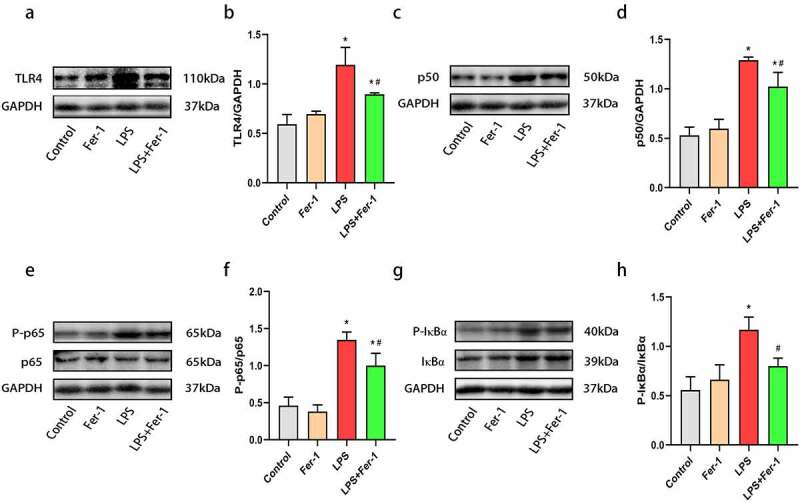
Fer-1 inhibited TLR4/NF-κB signaling pathway in septic rats. (a, b) Representative western blots of TLR4 in four groups of rats (n = 3 per group). (c, d) Representative western blots and statistical analysis of expression of NF-κB p50 in four groups of rats (n = 3 per group). (e, f) Representative western blots and statistical analysis of expression of NF-κB p65/P-NF-κB p65 in four groups of rats (n = 3 per group). (g, h) Representative western blots and statistical analysis of expression of P-IκBα/IκBα in four groups of rats (n = 3 per group). Data are expressed as mean ±SEM. *P < 0.05 vs. Control group, **#**P < 0.05 vs. LPS group

Abbreviations: Fer-1, Ferrostatin-1; TLR4, Toll-like receptor 4; NF-κB, Nuclear factor kappa B; IκBα, Inhibitor of kappa Bα.

Our previous investigations showed that TLR4 activation contributes to cardiac inflammation [20,22]. NF-κB is an important downstream inflammatory effector of TLR4 and is upregulated in the myocardium during sepsis [23]. In the present study, LPS markedly increased the levels of TLR4, NF-κB p50, P-NF-κB p65/NF-κB p65, and P-IκBα/IκBα, changes that was inhibited by Fer-1 (Figure 5(a-h)). In consequence, Fer-1 may relieve LPS-induced cardiac inflammation by inhibiting the TLR4/NF-κB signaling pathway.

## 4. Discussion

The major findings of the current study are described below. (1) Fer-1 improved cardiac systolic function and alleviated cardiac injury in LPS-treated rats. (2) Fer-1 inhibited LPS-induced ferroptosis in myocardium. (3) Fer-1 alleviated inflammatory responses in septic rats, and (4) Fer-1 inhibited the TLR4/NF-κB signaling pathway. Briefly, Fer-1 inhibited cardiac ferroptosis and inflammation, and then improve cardiac function at least partially by inhibiting the TLR4/NF-κB signaling pathway. Therefore, Fer-1 may regulate sepsis-induced cardiac dysfunction via the TLR4/NF-κB signaling pathway.

Sepsis is associated with multiorgan injury caused by systemic inflammatory response, in which cardiac dysfunction is a common manifestation [2,3]. LPS, a strong activator of sepsis, is commonly used to induce sepsis in animal models [24]. Based on the function of LPS, we used LPS to induce cardiac dysfunction. Sepsis-induced cardiac dysfunction was manifested by abnormal systolic function, specifically, a reduced LVEF (%) and LVFS (%) [19,24]. Consistent with a previous study, we found that LPS led to a reduced LVEF (%) and LVFS (%) and an increased LVEDV and LVESV, whereas treatment with Fer-1 significantly improved cardiac function. The serum levels of the enzymes CK-MB, LDH, and AST, which are major markers of cardiac injury, were measured to assess the severity of myocardial injury [25]. In this study, Fer-1 also significantly reduced the serum levels of myocardial enzymes that were increased by LPS.

Recently, ferroptosis was identified as one of the main pathogenic mechanisms of sepsis-induced cardiac injury [7]. During the development of ferroptosis, PTGS2 expression (a marker of ferroptosis) was increased [7,17]. In the present study, we found that PTGS2 was upregulated during sepsis, and Fer-1 treatment markedly decreased PTGS2 expression in the heart. Moreover, the accumulation of large amounts of lipid peroxidation products was reported as a key mediator of ferroptosis [15], and GPX4 deletion aggravated lipid peroxidation and caused ferroptosis [10,26]. Thus, we examined GPX4 expression in the myocardium, and found that LPS treatment resulted in lower GPX4 expression, which was restored by Fer-1. Consistent with the findings of the present study, Fer-1 also increased GPX4 expression in mice with high-fat diet-induced atherosclerosis [27]. These results suggest that ferroptosis is involved in LPS-induced cardiac dysfunction. Additionally, iron overload is regard as a central mediator of ferroptosis [17], and we also detected the expression of proteins associated with iron metabolism. Notably, ferritin is a key iron storage protein that is associated with iron homeostasis [11]. After LPS challenge, the heart tissues of rats showed elevated levels of FTL and FTH1. These changes are consistent with previous studies [28,29]. In mammalian cells, iron homeostasis mainly depends on iron transporters, such as FPN and TfR, among which FPN functions only as an iron exporter, whereas TfR is responsible for iron uptake into cells [11]. Previous studies have illustrated that a loss of FPN increases the intracellular iron level [30]. Park with colleagues found that increased TfR expression leads to iron accumulation, and then induces ferroptosis [31]. In the present study, Prussian blue staining and western blotting revealed significantly increased iron deposition in the heart after LPS administration, along with a decrease in FPN and TfR expression. However, iron deposition induced by LPS was reduced by Fer-1. Interestingly, TfR, an iron uptake protein, was decreased, while the iron accumulation in this study was upregulated. The possible reasons are the expression of FPN in LPS-induced heart were downregulated, resulting in iron overload. And compared with the decrease of TfR, the decrease of FPN may have a stronger effect on iron deposition. Taken together, these findings indicate that Fer-1 regulates ferroptosis in the myocardium during sepsis by inhibiting lipid peroxidation and iron overload.

LPS-induced inflammation plays a critical role in sepsis-induced cardiac dysfunction [7,19]. Indeed, we found that LPS promotes inflammatory cell infiltration and the expression of a series of inflammatory cytokines (including TNF-α, IL-1β, and IL-6) in the heart. Second, LPS increased systemic inflammation, which was characterized by increased serum TNF-α, IL-1β, and IL-6 levels, consistent with previous studies [19,24]. The deleterious effect was markedly decreased by Fer-1, indicating that ferroptosis may be an upstream event in the inflammatory response during sepsis. The mechanism of ferroptosis-mediated inflammation of the myocardium may be that ferroptosis promotes the release of DAMPs, which are in turn recognized by pattern recognition receptors, such as Toll-like receptors and Nod-like receptors, to eventually cause inflammatory responses [12,32]. Previous study has demonstrated that TLR4 knockdown improved cardiac remodeling by inhibiting ferroptosis [33]. It is widely believed that TLR4 plays a pivotal role in cardiac inflammation [13,22,34], and inhibition of TLR4 alleviates inflammatory responses in the heart [35]. In addition, NF-κB has been widely shown to function as a classical downstream mediator of TLR4 in the heart [21]. In the resting state, NF-κB is inactive status because it forms a trimer with its inhibitor IκBα in the cytoplasm. Upon stimulation, IκBα is phosphorylated and then promotes the translocation of NF-κB from the cytoplasm to the nucleus and phosphorylation, thus triggering inflammation and inflammatory cytokines release [36]. The levels of NF-kB and IκBα phosphorylation correlate with the severity of inflammatory responses [20,35]. Recently, chen with colleague reported that TLR4 activation is associated with ferroptosis in a heart failure model [33]. In the current study, we found that LPS challenge increased the levels of TLR4, NF-κB p50, P-NF-κB p65/NF-κB p65, and P-IκBα/IκBα, while Fer-1 exerted an anti-inflammation effect by reducing TLR4, NF-κB p50, P-NF-κB p65/NF-κB p65, and P-IκBα/IκBα levels. Thus, these results suggest that Fer-1 inhibited the activation of the TLR4/NF-κB signaling pathway, alleviating inflammatory responses caused by LPS stimulation.

## 5. Conclusions

Collectively, our study shows that Fer-1 improved sepsis-induced cardiac dysfunction and alleviated cardiac ferroptosis and inflammation which is at least partially by inhibiting the TLR4/NF-κB signaling pathway. Therefore, Fer-1 might represent a new target to prevent the development of cardiac dysfunction during sepsis.

## Highlights

1)Ferroptosis was activated in rats treated with LPS.

2)Ferrostatin-1 improved sepsis-induced cardiac dysfunction.

3)Ferrostatin-1 inhibited cardiac ferroptosis and inflammation.

4)Ferrostatin-1 may inhibit the activation of the TLR4/NF-κB signaling pathway during sepsis.
